# Gasoline Blow-Pipe

**Published:** 1890-11

**Authors:** J. J. Reed

**Affiliations:** Kansas


### GASOLINE BLOW-PIPE.

BY J. J. REED, KANSAS.

WHILE, perhaps, the largest proportion of dentists have facilities for soldering in the use of illuminating gas, there are still many who are compelled to make use of other and inadequate means to accomplish this work. The following description will, doubtless, be of use to this class.

The blow-pipe consists of a quart bottle with a mouth about one inch in diameter, fitted with a rubber-stopper, the latter perforated by two holes, through which pass two glass tubes, one extending nearly to the bottom of the bottle, the other merely passing through the stopper. Two rubber tubes, about four feet long, are attached to the glass tubes. To the end of one of these is inserted two inches of glass tubing to serve as a mouth-piece, and to the other the ordinary mouth blow-pipe. The bottle is half filled with gasoline.

By blowing through the mouth-piece tube, which should be attached to the tube extending to the bottom of the bottle, the air is made to combine with the volatile properties of the gasoline and passes out through the blow-pipe tube and may be ignited by an ordinary alcohol lamp. It produces an intense heat. If it is desired to increase the size of the flame, all that is necessary is to use blow-pipes of varying apertures from one-eighth inch down. Care should be used not to blow in the wrong tube, as this would force the gasoline out of the other tube. A foot blower will be found of advantage.

